# Supplementary material for: A whole-animal phenotypic drug screen identifies suppressors of atherogenic lipoproteins
Source: eLife. 2026 Aug 27;14:RP105314. doi: 10.7554/eLife.105314 (PMC13521566; doi:10.7554/eLife.105314)
Supplement: Supplementary file 2 [file elife-105314-code1.zip › JHDL_primary_result/Cupric chloride/Cupric chloride_JHDL_22_018.pdf]

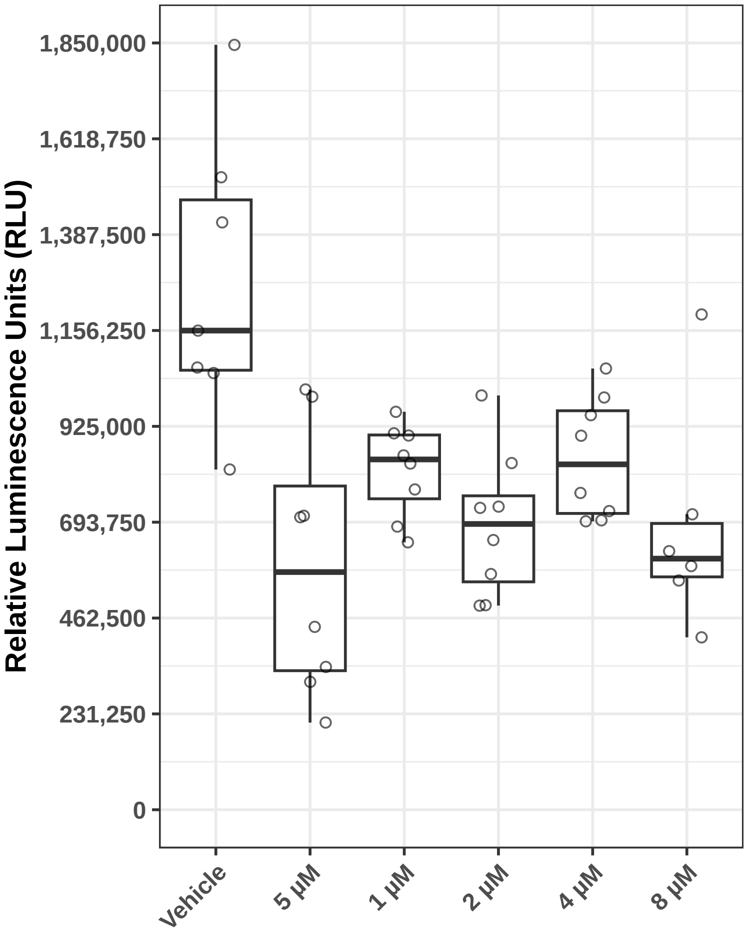

Relative Luminescence Units (RLU)
1,850,000
1,618,750
1,387,500
1,156,250
925,000
693,750
462,500
231,250
0
Vehicle
5 µM
1 µM
2 µM
4 µM
8 µM
